# Art Therapy With Jewish Ultra-Orthodox Children: Unique Characteristics, Benefits, and Conflicts

**DOI:** 10.3389/fpsyg.2020.598917

**Published:** 2020-10-27

**Authors:** Einat Doron

**Affiliations:** Faculty of Sociology, Adam Mickiewicz University, Poznań, Poland

**Keywords:** ultra-orthodox Jews, children, art therapy, secular therapist, inter-cultural therapy

## Abstract

The paper presents the potential benefits and conflicts of the encounter between Jewish ultra-orthodox (UO) children – belonging to a closed and segregated group – and art therapy – with its cultural, Western, secular, and professional characteristics. The paper describes the complex interface between the therapeutic use of art as a form of free expression and religious commandments and restrictions. The dialogue between art, therapy, and cultural religious boundaries is described through a case study of an 8-year-old UO boy and a secular female art therapist. Issues such as self-expression, gender roles, and identity exploration are discussed, emphasizing the unique characteristics, benefits, and conflicts of such an encounter.

## Introduction

Ultra-orthodox (UO) Jews constitute 11% of Israel’s Jews. They are characterized by their strict adherence to *halacha*, the Jewish law, which covers every aspect of daily life. The UO community have stringent standards of religious observance and rigid and conservative dress codes. UO communities tend to live in segregated neighborhoods with their own educational system so as to focus on religious precepts and protect themselves from secular influences (Witztum and Goodman, 1999). They maintain a cultural distance from (and often a hostility toward) the surrounding larger secular society. Rabbinically established standards of modesty dictate behavior and dress, including the covering of the lower neck, arms, legs, and, for married women, hair. Modesty laws are intended to avert any inappropriate out-of-wedlock attraction or objectification (Bachner-Melman and Zohar, 2019). UO Jews also distinguish themselves by speaking in Yiddish, a language used by Jews in central and Eastern Europe before the Holocaust so as not to desecrate the holy Hebrew language. Today, Yiddish is increasingly limited to their community (Heilman and Witztum, 1997). Television, cinema and the use of the internet for anything other than livelihood is forbidden. Smartphones with censored “kosher” internet use are allowed in some, but not all UO communities (Hoffman and Ben Shalom, 2011).

Practicing psychotherapy, with its Western origins, with children belonging to the UO community raises unique questions regarding treatment goals and intervention techniques. For example, the basic assumption of sharing, so fundamental in Western psychotherapy, is questionable for members of this community coming for treatment.

One of the difficulties experienced by UO children is a state of “selflessness.” This evolves due to what is termed the “lost children” syndrome in UO society, whereby less emphasis is placed on individual expression when children are raised in large families (Goren, 2014). The present paper discusses how the UO Jewish commandments and norms regard issues such as the expression of personal feelings and the exploration of the self in childhood.

Art as a method of therapy enables a projective dialogue which can shed light on these themes. A case study of an 8-year-old UO boy and a secular female art therapist illustrates how art therapy may address these very issues, both facilitating healing, while also evoking conflicts.

## Context

The case study took place in a children’s development center in Bnei Brak, the city in Israel most identified with the UO community. The children’s center offers paramedical treatments solely for UO children. The management and administrative staff were all from the UO community, while the therapeutic staff was mixed, secular and UO. When referred to a therapist, the parents were informed of the religious affiliation of the therapist. All the staff was required to dress according to the local modesty laws.

## Isaac

Isaac (pseudonym) was 8 years old when referred to art therapy by his physiotherapist, as she felt his progress was impaired due to emotional difficulties. Isaac’s mother agreed with this observation; she felt he was “not being normal,” acting childish and crying over every small incident. She said he felt he “was not loved.” His rabbi at school reported social difficulties and noted he was introverted and easily insulted. He described him as dreamy, moody and rated his overall functioning as below average. As the therapist felt his connection with his mother was a key factor in the therapeutic process, dyadic therapy was suggested. At first his mother was reluctant, saying he was the one in need of therapy. The therapist reflected to the mother the deep bond she saw between her and Isaac, as well as the strong impact she had on her son, arguing this could be used for Isaac’s benefit. She agreed to participate. In the dyadic therapy, she intervened frequently, leaving him little space and expecting things beyond his capacity. At times it seemed her interference was meant to prevent him from doing something wrong, awkward, or childish. In her presence, Isaac became quieter, allowing her to take the lead. He never confronted her. As often in therapy with UO children, the dyadic sessions ended abruptly, as the mother had to tend to her other many children. However, she did not end his therapy; perhaps because she was already seeing positive results, and also because the non-verbal projective nature of the intervention did not pose a direct threat to her cultural values. Once the mother was no longer present, Isaac went back to creating freely and with much investment. Due to the dynamic whereby Isaac tended to be subdued in his mother’s dominating presence, the therapist’s approach was to be as allowing and non-intervening as possible. Isaac was given complete freedom in his choice of materials. In the art therapy sessions, Isaac enjoyed working with fluid materials like acrylic. His works were idiosyncratic at first, in the sense that they were not understandable or communicative to the observer, and he often chose games intended for younger children. In the sessions, Isaac said he was unhappy at school as the rabbi hits if you make any mistakes. Thus, he never participated in the lessons. He felt as though the rabbi did not really know him and emphasized only cognitive functions and learning abilities. His parents assessed him according to the same criteria, but Isaac never directly talked about them. Through art and play, Isaac was able to express himself at his own pace. He revealed great curiosity, asking the therapist many questions regarding various topics and showed interest in the scientific aspects of materials and constructions. As his works became increasingly communicative and comprehensive to the observer, his mother reported less mood swings, wider social connections, and increased overall functioning.

As a secular art therapist, meeting Isaac’s therapeutic needs provided beneficial opportunities, while also raising many conflicts. Many of the therapeutic aspects involved inherent tensions between UO beliefs and what the therapist felt was necessary for Isaac to complete his therapeutic journey. The value of self-expression, prized, and cultivated in therapy, may be threatened outside of the safe container of therapy. Questions arose regarding Isaac’s ability to continue his inquisitive approach to the world as well as his ability to explore his own individuality. Would he be able to continue his journey of discovery without the actual artistic means of expressing himself? Would it not be dangerous to encourage something that later must be repressed? The case of Isaac offers a possible answer to these questions, as will elaborated in the discussion.

## Judaism and Psychotherapy

According to the UO perspective, psychology is perceived as a method that undermines the very fundamentals of religion, or even wishes to replace them with its own (Hoffman and Rossman, 2012). Western liberal perception gives priority to individual liberty and encourages values of independence and autonomy, whereas religion emphasizes obedience to the divine law and to rabbinical authority. Thus, the UO worldview does not encourage the values of independence and self-growth but rather tradition and conformity (Frosh, 2004). The UO value solidarity and collectivism, whereas psychotherapy enhances the individual and the self (Haimovich and Leiser, 2017). According to the UO worldview, the human psyche is to be controlled by religion (Caplan and Stadler, 2012). Thus, tension between issues of self vs. collectivism and individuality vs. conformity were salient in Isaac’s treatment, as will be elaborated in the discussion.

The characteristics of this ideological self-segregated group are gradually changing, as the UO community becomes increasingly established, self-confident and well-integrated into influential power positions in Israeli society. These significant changes have contributed to their increased willingness to embrace both the ideas and practices of the general Israeli culture, which until recently were perceived to be “meaningless, aimless, rootless, characterized by vandalism, crime, juvenile delinquency, the collapse of marriages and psychiatric disorders” (Caplan, 2006). One of the fields of Western knowledge that is being gradually assimilated is the field of psychological theory and practice (Caplan and Stadler, 2012). Over the past three decades, there has been a positive change of attitude toward disabilities, with a substantial decrease in stigma concerning mental health and treatment (Schnall et al., 2014); previous shame and concealment are being replaced with a deeper understanding of the need to address such issues (Haimovich and Leiser, 2017).

When seeking mental help, the leading rabbinic authority forbids psychiatric patients to seek assistance from a psychologist or psychiatrist who is a heretic or atheist (Rube and Kibel, 2004) due to the concern that non-orthodox practitioners may introduce ideas that violate religious-cultural norms and values. For example, patient and therapist may not share the same goals for treatment and thus may each have a different view of what constitutes “successful” therapy (Heilman and Witztum, 1997). However, these concerns do not align with the results of recent studies suggesting that religious (and non-religious) patients may benefit equally from treatment delivered by religious and non-religious therapists (e.g., Rosmarin and Pirutinsky, 2020). This gradual opening toward Western therapy is what enabled Isaac’s therapist to work in this children’s center, despite holding a secular worldview.

## Judaism and Art

Treating a patient from the UO community requires understanding some of the basic beliefs and norms this community holds regarding the self as well as their attitude toward art. The creation of art is associated with materialist fulfillment. This is in contrast to the expectation among the UO whereby fulfillment should be achieved through worshipping God and attention should be focused solely on the spiritual and not the material. The UO perspective aspires to maintain order and harmony, whereas art invites disorder. The creative endeavor encourages doubtful thinking whereas UO Judaism dictates subordination. Art by definition requires being involved in the self, whereas Judaism encourages letting go of one’s own petty world and investing in religion (Tauzinger, 2019). Nevertheless, UO parents allow children to create art, an activity that is unacceptable for adults. Thus, art therapy remains a successful choice for UO children. On one hand, it is not perceived as threatening to assimilate secular ideas in children, while on the other hand, it allows intrapsychic processes to occur through the artistic materials, facilitating change.

## Judaism and Male Attributes

As a fundamentally traditional society, the UO hold very distinct gender expectations. From a young age, these roles are taught and undisputed. Men are encouraged to focus their lives on studying Jewish texts; women are relegated to functions that support their husband’s spiritual development, such as earning money, and taking care of the home and children (Bachner-Melman and Zohar, 2019). Judaism stresses behavior over psychology, and responsibility over identity (Cashman, 2015). Men are expected to be gentle and to avoid violent confrontations with other men.

For children, these expectations are instilled whereby boys are destined to be “learners” and girls are “tsedeykeses” (righteous women) (Benor, 2004). The UO boy is thus valued according to his learning skills. The school he attends indicates an important aspect of his future, and yeshivas can be classified according to the degree of restraint, control, and self-denial required of their students (Hakak, 2009). For UO boys the hero is not the conqueror of fortresses and vanquisher of enemies, but “he who conquers his passions,” particularly as expressed through the study of Torah (Hakak, 2009). Feelings such as pride, anger, and indulgence are considered negative manifestations with which one should struggle. All the more so sexuality, aggression, and desire. These and other so-called negative emotions must be “worked on” (Hakak, 2012). Isaac, having to conform to this ideal male image, could never be the hero his parents expected of him. He was also unable to nurture the hero he wanted to become, as will be elaborated in the discussion.

## Expression of Feelings

Open expression of anger is viewed as unacceptable and is actively discouraged by the UO. Children may learn to avoid the direct communication of feeling and as adults fail to act using constructive methods of conflict resolution. Instead, they may struggle to maintain a conflict-free appearance (Wieselberg, 1992). When the child is perceived as difficult by his parents, fathers may be subsequently led to display less acceptance toward him or her (Finzi-Dotan and Gilerenter, 2018). As mentioned above, boys are valued according to their learning skills and performance. Isaac was below average, which was a source of concern for his parents.

The obligation to revere and honor one’s parents is fundamental to Judaism and is included in the first five commandments. Biblical commentaries explain the identification of respect for one’s parents with respect for God. Parents are, therefore, the means of bringing God into family life and their authority is a prerequisite for the authority of tradition (Wieselberg, 1992). Hierarchy within the family, reflecting rules of power, is also religiously and socially sanctioned. The parental subsystem is expected to be highly authoritarian with clear executive functions in relation to the child subsystem (Finzi-Dotan and Gilerenter, 2018). Early in therapy the familial hierarchy must be addressed, with recognition of parental authority. Careful tracking of eye contact will usually reveal who is the authority that allows children to speak. The commandment “Honor thy father and thy mother” (Exodus 20:1–21) must be acknowledged and parents (particularly fathers) must give explicit permission to the children to describe what transpires at home (Wieselberg, 1992). A second important commandment instructs against the use of *lashon hara*, “negative talk.” This means it is forbidden to speak negatively about someone else, even if it is true. “Whoever of you desires life… guard your tongue from evil” (Psalms 34:12–13). Even recounting a negative event without using names is discouraged, for the listeners might be able to figure out the persons involved (Chafetz Chaim 3:4). In a therapeutic setting, these two commandments may paralyze children into silence, or prevent parents speaking about one child in the presence of another (Wieselberg, 1992). Moreover, some UO Jews do not wish to be treated by psychotherapy but with medication alone in order to assure the avoidance of discussing conflicts or the risk of expressing anger that may give the appearance of dishonoring one’s parents or one’s heritage (Rube and Kibel, 2004).

While Western culture, influenced by consumer culture, stresses dress, and external appearance as central for self-expression and self-realization, in the UO society, adhering to the UO dress code is a manifestation of one’s commitment to God and the community (Hakak, 2009). Boys have a strict and uniform dress code and it is a common sight to see all the girls of a family dressed identically. The impossibility of presenting one’s self through physical appearance is just one example of how expression of self-identity is discouraged.

## Discussion

Due to the stigma about mental health and behavioral problems in the UO population, one of the results is a preference to send their children to an art therapist rather than to a psychologist. Even though the religious authorities instruct to seek care within the UO community, it was the therapist’s experience that they sometimes felt more comfortable with her as a secular therapist because she could not recognize the family and thus issues raised in therapy could not cause potential future damage to the family’s reputation. It was also her experience that mothers often felt more comfortable confiding in her and allowing themselves to be heard in a manner they would not have voiced otherwise.

The following discussion will elaborate how the aforementioned aspects and beliefs of the UO way of life influence the therapeutic process, while highlighting how the unique characteristics of the arts serve as a beneficial treatment method in light of these factors.

### Art as a Common Language

Western therapeutic traditions have often reflected and reinforced the status quo with their assimilationist and ethnocentric modus operandi. Art therapy provides an opportunity to respond differently through self-critique and scrupulous attempts to be a progressive and culturally sensitive enterprise (Hocoy, 2002). Inter-cultural therapy calls for a modification of the strategies for interaction. These modified strategies require both therapist and patient to find some common cultural ground on which to meet in order to enable them to “speak some common language” (Heilman and Witztum, 1997). Despite its Western origins, art therapy is regarded by some (e.g., Kalish-Weiss, 1989) as being less culture bound than other therapeutic modalities as it is less encumbered by linguistic expression. Furthermore, art is a form of communication where the artist communicates with his or her audience in a symbolic language that is commonly understood (Kramer, 2000). The role of art can offer a child an alternative means of communication which does not involve sophisticated speech. Instead, it offers the child another language, nonverbal and symbolic through which feelings, wishes, fears, and phantasies can be expressed (Case and Dalley, 1989). Sometimes it is enough for the child to create the artwork and few words are exchanged (Waller, 2006). This serves as a key factor as some of the UO children do not speak fluent Hebrew and art therapy utilizes art as a form of common communication lessening the barrier of language differences.

### Art as Sublimation

Making art in the safe container of therapy may enable a child to get in touch with feelings that cannot easily be expressed in words; pain, rage, shame, and other difficult feelings are directed into the artwork which can then be shared with the art therapist and encourage positive change (Waller, 2006). The reflection of the self through art may be less intimidating and may assist individuals who are threatened by intimate engagement to continue exploring their emotions (Springham et al., 2012). The abstract framework of symbols and conventions in creating art enables a distance from reality and thus allows the child to enjoy the artwork without guilt or anxiety from what is being reflected or due to the reaction of the child’s surroundings. Through art a child may communicate their inner experiences in a sublimated, culturally and socially productive form. An object may emerge from destructive and aggressive feelings which can symbolize those feelings and thus prevent the patient from either internalizing them in unhealthy ways or acting them out in destructive ways (Kramer, 1971, 2000; Saunders and Saunders, 2000).

For UO children who are discouraged from showing emotions, and particularly negative emotions, this expressive yet indirect channel serves as an important opportunity. As a projective tool, art therapy allows UO children to openly express themselves without the fear of being disrespectful to their parents, without the fear of “lashon hara,” without the fear of breaking norms or harming their reputation as being a “good girl/boy.” Thus, art therapy’s indirect channel of expression serves as a key factor for all children, but is particularly significant for children from the UO community who are expected to silence and renounce negative feelings. For secular children, these feelings are usually contained to a certain extent, even if not encouraged. In Isaac’s case, he had many negative feelings arising in him. He could say he did not feel loved, but he could not freely express his fear of the rabbi, nor his frustration with his parent’s attitude. He even allowed his mother to feed him at the late age of seven, just to avoid her getting upset with his dawdling during lunch. The battle scenes in his artwork allowed him to “fight” the situation and express aggression in a sublimated manner.

When a child becomes deeply immersed in the physical process of painting it enables a loosening of control (Waller, 2006). For UO children this is a major benefit, for their everyday life is characterized by an expectation of self-restraint. The sessions with Isaac were a legitimate space for letting go; he no longer had to conform himself to fit the framework. The physical involvement in exploring art materials appears to be of great benefit to children. The physicality of the engagement with the art materials, the activation of the senses and the movement that occurs, triggers both body and mind (Czamanski-Cohen and Weihs, 2016). Some art therapists focus on the physical enjoyment, and the “play” elements of art therapy, as contributors for psychological growth (Case and Dalley, 1989). Working with clay for that matter, requires active involvement of the body. Aggressive regression is common in children, where violence and anger may be expressed through hand movements or with the use of clay tools in aggressive ways, such as stabbing, deep cutting, scratching, clasping, throwing, and smashing (Sholt and Gavron, 2006). Two-dimensional materials may be torn, cut, ripped, or crumpled. All of these options offer the child methods of sublimation that are expressive yet legitimate.

Isaac’s works involved three main themes: (1) maritime themes, dividing the paper into two worlds- above and beneath the surface of the water; (2) battle themes: scenes describing battles, usually in the water, including pirates and water creatures. Isaac was able to be the winner, powerful, and heroic; and (3) clay, which Isaac particularly enjoyed working with, investigating its qualities and constructive features. The two-dimensional worlds of his paintings transformed into clay kingdoms, with warrior figures, weapons, and an impressive clay fortress, replete with torches containing “fire” made from plasticine. His dreamy character, as described by his rabbi, now transformed into a rich imagination and fascinating stories. Isaac could obtain strength through play and art and proudly display his hidden talent as an artist and a constructor.

Art therapy allows children to play and make-believe, to dress up and act. A boy can become a hero by wearing a cape and “saving his friend,” by being the race driver and “rushing to the scene of the accident.” In UO society, boys are appreciated for their ability to memorize versus. Art therapy allows children to play diverse roles and explore various and additional ways of expressing their tendencies or feeling heroic. Isaac used to build constructions and make experiments with artistic materials, assessing their qualities. He clearly had the soul of a scientist. Yet it was unclear whether these qualities would be appreciated outside the therapy room. Art therapy enables children to play in a non-subordinated manner and free of judgment. Hopefully Isaac could translate his newfound curiosity into culturally accepted avenues of expression.

### Art as a Means of Exploring Individuality

Children in the UO community have very little personal choice; every aspect of their life is predetermined and chosen for them, including their dress code, what they study, their future occupation, and in many cases, whom they will marry. Personal opinions are not considered a virtue but rather something to suppress. Art therapy not only allows but requires the ability to make choices. Engaging in such therapy requires an opinion and being active, as each session entails making many choices, such as what material to work with, what size paper, what color paper, which paintbrush, and which shades to use. When working with UO children, letting them decide for themselves is a major therapeutic objective.

Whereas UO children are constantly required to choose between right and wrong within a rigid structure (Wieselberg, 1992), art therapy offers many options of middle shades and flexible structures, which help develop useful coping mechanisms.

The exploration of identity often discloses contradictory aspects of the self. This is particularly important for UO children as they are exposed to a myriad of opportunities in the art therapy sessions. Relating to these opposites in the artwork enables integrating conflicting aspects in the personality. Examples include relating to the inside of a clay sculpture as opposed to its outside, or a soft or smooth texture in contrast to a rough texture. Working with clay (like all artwork) offers the opportunity to represent images that are distorted, intimidating, or ugly (Sholt and Gavron, 2006). By facing these qualities in their creation, the child, with the support and mediation of the therapist, can then accept these qualities in themselves.

According to Western perspectives, Isaac’s mother was highly controlling. She intervened in many aspects of the session, not allowing him to make any independent decisions. Even in light of expected norms of parental authority and obedience by children in the OU community, the extent of her interference and her candid lack of trust in her son, were prominent and exceptional. It was the therapist’s task to find a path in which Isaac could explore his wishes and express them without challenging his mother’s authority. In addition, the importance of this had to be delicately explained. It had to remain sensitive to what extent comment could be made on what is perceived by Western culture as invasive behavior by his mother, without undermining her authority. Was Isaac’s reluctance to confront due to his fear of losing the object’s love or out of religious values, or perhaps a combination of both?

Working with Isaac raised many questions regarding the above issues. The two worlds described in Isaac’s works above and below water signified contradictory aspects of his personality, which was enhanced by the therapeutic process. As a secular art therapist working in an UO setting, the therapist faced the dilemma of whether I should answer his scientific questions, or should I give the expected answer that everything has been made by God? Another question was how to mediate Isaac’s tendencies to his parents in a way that would encourage them to help him develop these tendencies and connect to his strengths. Art therapy can enable seeking an inner voice and opening a path to becoming one’s unique self. If mediated correctly and put in a therapeutic context, the process occurring within the framework of art therapy will most likely not pose a threat to the UO family, that is extremely concerned with the responses of the wider community. The mediation process with the parents was most successful when the therapist concentrated on presenting Isaac’s strengths which could serve him outside the room, such as in his studies through what is termed “hitpalpelut” – Jewish debating, which requires creativity through personal insights to Jewish texts. Isaac’s inner conflicts were not elaborated with the parents. Witnessing her son gain confidence and self-assurance, allowed Isaac’s mother to view him in a more positive manner. The therapeutic process enabled him to connect to his inner world and build his sense of self that was so lacking. This new foundation gave him the confidence to communicate better both at home and in school, despite not being able to directly express certain aspects of himself out of the sessions. He now felt as a significant member of his family, and his mother was clearly proud of him and testified that he dramatically changed. Art allowed a sublimated approach to intrapsychic processes, yet more importantly, the therapeutic process enabled the creation of a “potential space” (Winnicott, 1971/1991) where within the safe interpersonal field of therapy Isaac could connect to his playfulness and discover his authentic self. This authentic self was something that he could own and rely upon and that persisted outside of therapy as well. His mother noted this and appreciated his increased abilities at home and in school. Perhaps the bridge between the Western emphasis on individuality and the cultural norm of self-repression in UO society was the mother’s true wish to see her son’s increased sense of aliveness. This sense of aliveness need not be specifically expressed through art; it may simply be the ability to speak out, and to feel worthy of being heard and loved. Isaac improved in these aspects without creating any conflict in his surroundings.

## Future Implications

The unique characteristics and benefits of art therapy with children from the UO community can also be translated and applied to members of other fundamental societies both in Israel and elsewhere. In places where secular therapists who hold a worldview based on Western values interact with clients from societies that value conformity and self-repression, bridges must be built through which to foster trust and openness to the experience of therapy. As a non-verbal mode of communication, art therapy offers a unique bridge between such cultures. At the same time, the points emphasized above may enrich and stress the benefits of using art as a form of therapy also by a therapist from within the OU community. These therapists have recently become more prevalent due to processes of change in the UO population that have opened opportunities for pursuing a career in art therapy.

## Data Availability Statement

The original contributions presented in the study are included in the article/supplementary material, further inquiries can be directed to the corresponding author.

## Ethics Statement

Ethical review and approval was not required for the study on human participants in accordance with the local legislation and institutional requirements. Written informed consent to participate in this study was provided by the participants’ legal guardian/next of kin. Written informed consent was obtained from the minor(s)’ legal guardian/next of kin for the publication of any potentially identifiable images or data included in this article.

## Author Contributions

The author confirms being the sole contributor of this work and has approved it for publication.

## Conflict of Interest

The author declares that the research was conducted in the absence of any commercial or financial relationships that could be construed as a potential conflict of interest.
